# Acquired von Willebrand Disease Secondary to Clear Cell Renal Cell Carcinoma

**DOI:** 10.1089/cren.2018.0032

**Published:** 2018-07-01

**Authors:** Brian Odom, Iyad Khourdaji, Victoria Golas, Richard Zekman, Bradley Rosenberg

**Affiliations:** ^1^ Department of Urology, Beaumont Health, Royal Oak, Michigan.; ^2^ Department of Urology, University of Virginia, Charlottesville, Virginia.; ^3^ Department of Hematology and Medical Oncology, Beaumont Health, Royal Oak, Michigan.

**Keywords:** acquired von Willebrand disease, renal cell carcinoma, laparoscopic nephrectomy, nephrectomy, clear cell carcinoma, von Willebrand disease

## Abstract

***Background:*** Acquired von Willebrand disease (AvWD) is a rare and often underdiagnosed disease that typically is associated with lymphoproliferative, cardiovascular disease, and myeloproliferative disease. It is challenging to diagnose as it requires a hemostatic challenge to present itself.

***Case Presentation:*** This is a 46-year-old male with a history of multiple sclerosis complicated by neurogenic bladder who presented with intermittent gross painless hematuria. He underwent a gross hematuria workup. Cystoscopy demonstrated active bleeding from the right ureteral orifice. CT Urogram showed a filling defect in the right renal pelvis and endophytic 3 cm solid, enhancing left kidney mass. The patient underwent diagnostic cystourethroscopy, bilateral retrograde pyelogram demonstrating no filling defects bilaterally. Right ureteropyeloscopy demonstrated diffuse patchy erythema of the infrarenal collecting system with biopsies obtained. His postoperative course was complicated by gross hematuria requiring cystoscopy which demonstrated no upper tract bleeding and small pulsatile bleeding vessel in the bladder requiring cauterization. Hematology was consulted to rule out bleeding diathesis with workup demonstrating a von Willebrand deficiency (vWD). He had no family history of vWD and an AvWD was suspected. Hematologic workup was consistent with AvWD, type 2B vWD also known as a platelet-type von Willebrand disease. Renal pelvis biopsies were negative for pathology. Further investigation of the left renal mass confirmed a biopsy-proven clear cell renal cell carcinoma (ccRCC). He underwent a laparoscopic left radical nephrectomy with final pathology demonstrating pT1 ccRCC with negative margins. Postoperatively his repeat laboratories demonstrated normal factor VIII activity, ristocetin cofactor, and vWF antigen with normalized activated partial thromboplastin time. Follow-up imaging demonstrated no further evidence of disease supporting the hypothesis of a paraneoplastic syndrome from his ccRCC that caused an AvWD.

***Conclusion:*** This is the first case report to our knowledge of a paraneoplastic AvWD secondary to ccRCC. This should be on your differential when there is abnormal bleeding in the setting of renal masses.

## Introduction and Background

Acquired von Willebrand disease (AvWD) is a rare but potentially catastrophic hematologic disorder that is often undiagnosed. The challenge in detecting this bleeding condition is that milder forms of AvWD do not typically present until instigated by a hemostatic challenge.^1^ In the context of the present case report, an underlying bleeding disorder was suspected because of severe bleeding with no obvious underlying cause.

## Presentation of Case

**FIG. 1.**
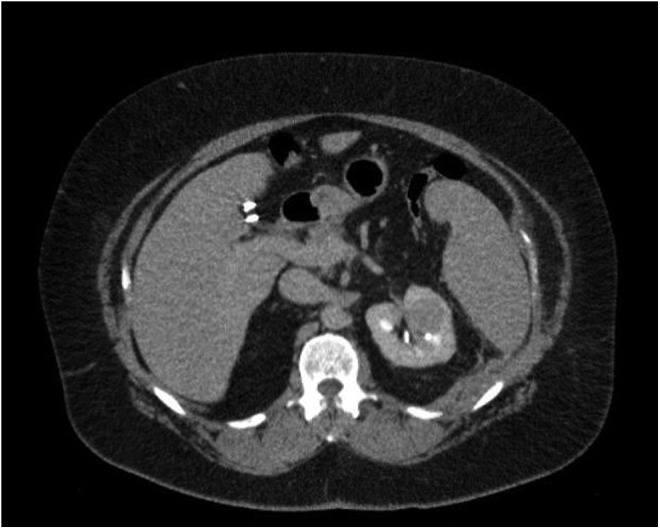
CT scan demonstrating 3 cm enhancing left renal mass.

A 46-year-old male with a past medical history of multiple sclerosis (MS) presented with a few-week history of intermittent gross painless hematuria. In the office, cystoscopy was performed and demonstrated active bleeding from the right ureteral orifice. CT urography was performed at that time identifying a 1 cm filling defect involving the right renal pelvis. Furthermore, the CT scan also identified a completely endophytic 3 cm solid, enhancing mass involving the left kidney (Fig. 1). The patient was subsequently referred to our clinic for further management.

The patient's past urological history was significant for urinary tract infections and incontinence secondary to a neurogenic bladder from MS. His voiding habits are aided with Silodosin. The patient otherwise had no personal history of genitourinary malignancy or nephrolithiasis. Moreover, he denied any previous history of smoking or chemical exposures. He had no family history of genitourinary malignancy. His physical examination was significant for obesity, but was otherwise unremarkable. The patient's baseline kidney function was within normal limits.

Cystourethroscopy and bilateral retrograde pyelograms were performed to investigate the aforementioned hematuria and proximal right renal pelvic filling defect. Bilateral retrograde pyelograms demonstrated no discrete filling defects. Right ureteropyeloscopy using initially a semi-rigid and eventually flexible ureteroscope with a 12 to 14 access sheath was performed identifying severe, diffuse patchy erythema of the intrarenal collecting system of which representative biopsies were obtained, but no lesions were definitively suspicious for transitional cell carcinoma. His postoperative course was complicated by severe gross hematuria requiring a return to the operating room. Cystoscopy demonstrated no more upper tract bleeding, but a tiny pulsatile bleeding vessel located in the bladder near the right ureteral orifice, which was cauterized. Ultimately, pathology from his renal pelvic biopsy was negative for malignancy.

Hematology consultation was sought to rule out a bleeding diathesis. The patient was found to have a prolonged activated partial thromboplastin time (aPTT) of 40.6 seconds with a normal PT time and Thrombin Time. The work-up for the prolonged aPTT included a mixing study, a Factor IX activity level, a Factor VIII activity level, and a vWF antigen. He was found to have an increased Factor IX activity level (143%) with repeat within normal limits (108%) drawn 1 day after the initial. His Factor VIII activity level was decreased (13%) and he had a decreased vWF antigen (<15%). His ristocetin cofactor was decreased at <20%. This raised a concern for vWF deficiency. Given lack of prior bleeding and no family history, an acquired von Willebrand syndrome was suspected.

An autoimmune work-up, including testing for systemic lupus erythematosus and antiphospholipid testing were within normal limits. Thyroid testing was within normal limits. The serum protein electrophoresis showed no monoclonal paraprotein disorder. Workup for anemia was consistent with blood loss.

The patient's factor VIII activity level at that time was decreased (36%) with decreased vWF antigen level (29)% and vWF activity level (21%). The von Willebrand multimer testing was performed to further differentiate the type of vWF deficiency. Testing revealed loss of the very highest molecular weight multimers consistent with underlying acquired vWF syndrome, platelet-type von Willebrand disease or type 2B von Willebrand disease. Platelet function analysis was abnormal, thought to be due to vWF deficiency. The patient was started on a trial of Desmopressin (DDAVP) nasal spray. It was noted that the Factor VIII levels did not rise with DDAVP on repeat testing.

Simultaneously, further investigation was initiated on the left renal mass, which was biopsied under CT guidance and found to be consistent with nuclear grade 2 clear cell renal cell carcinoma (ccRCC). It was thus proposed that the patient had acquired von Willebrand Disease secondary to a paraneoplastic syndrome from RCC. He was subsequently advised to undergo left radical nephrectomy.

Before the nephrectomy and 3 days postoperatively, the patient was started on Humate and intravenous immune globulin infusion. Subsequently, improvement of vWF antigen level (57%) and ristocetin cofactor level (32%) was noted. The aPTT level was 29.8 seconds and Factor VIII activity level was 79% after Humate and IVIG. He subsequently underwent an uncomplicated laparoscopic left radical nephrectomy. Three days after nephrectomy, the patient had Factor VIII level of 270%, ristocetin cofactor >150%, and vWF antigen level at >300%. Seven days postoperatively, patient had Factor VIII levels that had normalized (131%). Hemoglobin and coagulation studies all remained within normal limits during his hospital course. He was ultimately discharged home on postoperative day 4 in a stable condition. Final pathology from the nephrectomy revealed a pT1a ccRCC, Fuhrman nuclear grade 2 with negative margins.

A couple of months after the nephrectomy, the patient's repeat laboratories demonstrated normal factor VIII activity (84%), ristocetin cofactor (65%), and vWF antigen (76%) levels with normalized aPTT. The patient had no evidence of disease on CT abdomen/pelvis supporting the hypothesis that the patient had a paraneoplastic syndrome from his renal cell carcinoma that caused an acquired von Willebrand disease.

One year following nephrectomy, he was admitted to the hospital for dehydration and malaise and was found to have palpable right supraclavicular lymphadenopathy with CT of the neck with IV contrast demonstrating bilateral cervical adenopathy. He underwent biopsy, which demonstrated a chronic lymphocytic lymphoma/small lymphocytic lymphoma that has not required treatment. Cervical adenopathy was not present at the time of von Willebrand workup or laparoscopic radical nephrectomy and it is unlikely that indolent lymphoma is the etiology of his von Willebrand disease. It should be noted that the patient has not had a recurrence of his von Willebrand disease since nephrectomy.

## Discussion

Most cases of AvWD have been associated with lymphoproliferative disorders (48%) followed by cardiovascular disease (21%), and myeloproliferative diseases (15%).^2^ AvWD has been reported with nonhematologic neoplasms such as Wilms' tumors, Ewing's sarcoma, and peripheral neuroectodermal tumors and more rarely described in bladder and prostate cancers.^2–4^

Several pathological mechanisms have been proposed for AvWD, including specific or nonspecific antibodies to vWF, aberrant tumor expression of GP1b or GP IIb/IIIa receptors resulting in absorption of vWF, increased proteolytic degradation of vWF, or loss of high-volume vWF multimers under shear stress.^1,5^ Diagnosis requires the absence of personal or family history of bleeding with findings typical of von Willebrand deficiency (vWD; reduced vWF antigen, vWF:RCo, and vWF/Co:antigen ratio).^2^ Patients typically present with mild to moderately severe mucocutaneous bleeding similar to inherited vWD; however it may remain occult in milder forms of AvWD.^2^

To our knowledge this is the first case of paraneoplastic AvWD reported in ccRCC. Associated paraneoplastic syndromes occur in 10% to 40% of patients with RCC.^6^ More commonly these are constitutional symptoms (fever, anemia, hypertension, or cachexia); however hypercortisolism, hypercalcemia, polycythemia, and liver dysfunction have been reported.^6^ Clinicians must consider acquired hematologic disorders in patients presenting with renal masses and signs of abnormal bleeding. At 3 months postoperatively, this patient has no evidence of metastatic spread and complete resolution of AvWD following laparoscopic nephrectomy.

## Conclusion

This case, to our knowledge, is the first report of paraneoplastic AvWD reported in ccRCC. It should be in a clinician's differential when patients develop signs of acquired hematologic disorders in the setting of renal masses and signs of abnormal bleeding.

## Abbreviations Used

aPTT: activated partial thromboplastin time
AvWD: acquired von Willebrand disease
ccRCC: clear cell renal cell carcinoma
CT: computed tomography
DDAVP: Desmopressin
MS: multiple sclerosis

## References

[1] Shetty S, Kasatkar P, Ghosh K Pathophysiology of acquired von Willebrand disease: A concise review. Eur J Haematol 2011;87:99–106 21535159 10.1111/j.1600-0609.2011.01636.x

[2] Federici AB, Rand JH, Bucciarelli P, et al. Acquired von Willebrand syndrome: Data from an international registry. Thromb Haemost 2000;84:345–349 10959711

[3] Dumas G, Rousseau B, Rodrigues MJ, Tlemsani C, Goldwasser F Acquired Type II von Willebrand syndrome in locally advanced bladder cancer successfully treated with intravenous immunoglobulin and chemotherapy. Clin Genitourin Cancer 2016;14:e95–e97 26362071 10.1016/j.clgc.2015.07.013

[4] Claus PE, Van Haute I, Verhoye E, Deeren D, Moreau E Diagnostic challenges in acquired von Willebrand syndrome: A complex case of prostate carcinoma associated-acquired von Willebrand syndrome. Semin Thromb Hemost 2017;43:101–104 27806384 10.1055/s-0036-1592167

[5] Federici AB, Budde U, Rand JH Acquired von Willebrand syndrome 2004: International Registry—Diagnosis and management from online to bedside. Hamostaseologie 2004;24:50–55 15029273 10.1267/hamo04010050

[6] Sacco E, Pinto F, Sasso F, et al. Paraneoplastic syndromes in patients with urological malignancies. Urol Int 2009;83:1–11 19641351 10.1159/000224860

